# Use of Genetic Data to Infer Population-Specific Ecological and Phenotypic Traits from Mixed Aggregations

**DOI:** 10.1371/journal.pone.0098470

**Published:** 2014-06-06

**Authors:** Paul Moran, Jeffrey F. Bromaghin, Michele Masuda

**Affiliations:** 1 Conservation Biology Division, Northwest Fisheries Science Center, Seattle, Washington, United States of America; 2 United States Geological Survey, Alaska Science Center, Anchorage, Alaska, United States of America; 3 Auke Bay Laboratories, Alaska Fisheries Science Center, Ted Stevens Marine Research Institute, Juneau, Alaska, United States of America; Centers for Disease Control and Prevention, United States of America

## Abstract

Many applications in ecological genetics involve sampling individuals from a mixture of multiple biological populations and subsequently associating those individuals with the populations from which they arose. Analytical methods that assign individuals to their putative population of origin have utility in both basic and applied research, providing information about population-specific life history and habitat use, ecotoxins, pathogen and parasite loads, and many other non-genetic ecological, or phenotypic traits. Although the question is initially directed at the origin of individuals, in most cases the ultimate desire is to investigate the distribution of some trait among populations. Current practice is to assign individuals to a population of origin and study properties of the trait among individuals within population strata as if they constituted independent samples. It seemed that approach might bias population-specific trait inference. In this study we made trait inferences directly through modeling, bypassing individual assignment. We extended a Bayesian model for population mixture analysis to incorporate parameters for the phenotypic trait and compared its performance to that of individual assignment with a minimum probability threshold for assignment. The Bayesian mixture model outperformed individual assignment under some trait inference conditions. However, by discarding individuals whose origins are most uncertain, the individual assignment method provided a less complex analytical technique whose performance may be adequate for some common trait inference problems. Our results provide specific guidance for method selection under various genetic relationships among populations with different trait distributions.

## Introduction

The widespread implementation of highly polymorphic genetic markers has opened important new avenues in ecological genetic studies. For example, it is now possible using genetic stock identification (GSI) to accurately allocate a mixture of unknown individuals to probable source populations, either as individuals or as modeled proportions by using a baseline data set of genetic information from individuals of known origin [1], [2]. The power of these methods is remarkable and is generally only limited by the discriminatory power of the reference baseline to which unknown individuals are compared.

There is much current interest in assignment of individuals to population of origin [3], and this new suite of powerful genetic tools has gained widespread ecological utility in the research and management of Pacific salmon (*Oncorhynchus* spp.). These studies are possible because of the availability of powerful, range-wide, genetic baseline data. Numerous genetic baselines have been developed to support mixed fishery applications for harvest management. For example, a large interagency collaboration, the Genetic Analysis of Pacific Salmonids (GAPS) consortium, recently produced a shared, standardized, microsatellite baseline, data set for Chinook salmon (*O. tshawytscha*) that nearly covers the species range [4].

The availability of these genetic baseline data sets has sparked considerable interest among ecologists and oceanographers who recognize that genetic information from mixed fishery samples can be used to infer population-specific ecological traits among sources. Most applications of individual assignment (IA) relate to study of genetic structure, connectivity and estimation of population genetic metrics and parameters [5]. However, more novel ecological applications include investigation of body burden of ecotoxic compounds [6], parasite loads [7], [8], ELISA or qFAT values [9], and habitat use [10]. These methods have even found use in killer whale (*Orcinus orca*) trophic studies of predation on specific Chinook salmon populations [11]. Hereafter we refer to “ecological or phenotypic trait” or simply “trait” to identify the specific characters we studied (Chinook salmon fecundity and disease prevalence). A diverse array of possible character traits are amenable to our approach. Our goal was not to relate genotype to phenotype. Rather, we used genetic information to infer population of origin and then estimated the distribution of the phenotypic trait (genetic or non-genetic) in the reporting groups of interest (where a “reporting group” is an aggregate of genetically similar biological populations [4], see Materials and Methods).

The current general approach of trait inference studies is to sample individual animals in mixed-population aggregates, use genetic information to assign individuals to source populations (for which their posterior probability is maximal), and then treat their ecological traits as a representative sample of those traits in in the reporting groups contributing to the mixture, e.g., [12]. Some ecological or phenotypic traits, such as fecundity, can be directly studied by sampling individual populations; however, time and resources can be saved by inferring the traits from mixture samples already collected and genotyped as part of routine harvest and bycatch management. There is also sometimes the goal to study traits in life stages where animals are in mixed population aggregates (e.g., during dispersal or migration). Given the increased use of genetic data for this purpose, investigation is warranted into the utility of more powerful analytical alternatives that use more of the available information and more realistically incorporate uncertainty, e.g., [13].

Despite the significant power provided by many contemporary genetic baseline data sets, (e.g., the GAPS-Chinook, microsatellite baseline [4]) uncertainty in IA remains a thorny issue for studies of this kind. In most studies, individual animals have excellent data quality (typed for all or nearly all loci, i.e., little missing data) and assign with high probability to their putative population of origin. However, some fraction of individuals may have missing data for some loci, low assignment probabilities, or both, and there is always some assignment error, even among highly distinct populations. In these cases, investigators sometimes omit individual fish with these ‘problems’ by, for example, removing fish typed for fewer than some number of loci, or whose highest assignment probability to reporting group is below 0.8 [9], [12] or some other predetermined value. The logic is that individuals with uncertain origin – for whatever reason – will reduce estimation accuracy and precision. Moreover, individuals that fail to produce reliable genotypes for most loci probably reflect poor tissue quality resulting in degraded DNA and less confidence in the few genotypes that are produced. This filtering should still produce unbiased mixture proportion estimates if omitted individuals are randomly distributed among potential source populations. That assumption is probably valid for individuals with poor data quality in mixture studies, i.e., animals that fail to genotype are not likely to come predominantly from a single population. Instead genotyping failure is generally due to degraded, low-molecular-weight DNA, PCR inhibitors, etc. and is more likely related to collection of the mixture sample, rather than the source populations that contribute to the mixture. Even so, discarding data reduces sample size and potentially estimation precision. Moreover, it would seem desirable to retain samples that have high assignment probability despite being typed for few loci, as long as those few genotypes are confidently scored.

The omission of individuals with uncertain assignment can bias estimates of mixture composition and potentially trait estimates as well. The relative probabilities of assignment are in part a function of how reporting groups are defined. The reporting groups are higher-level aggregates of populations. IA or fractional allocation (see below) is first made at the level of populations, and then those assignment probabilities are combined to the higher-level reporting groups (e.g., “stocks” in fisheries, “management units” in wildlife, etc.). Bias will be minimized when the reporting groups closely reflect the patterns of genetic relatedness among populations. However, research hypotheses or management questions may require genetically similar stocks to be placed in different reporting groups. Reporting groups that are not clearly distinguished genetically result in lower probabilities of assignment to true reporting group of origin because assignment probability may be split between genetically similar reporting groups. Those split probabilities can result in omission of that individual fish if no single assignment value exceeds the minimum probability threshold, e.g., 0.8. Even if the reporting groups were genetically distinct, the distribution of assignment probabilities will differ among stock groups, and omission of individuals on that basis could bias stock composition estimates. This bias may or may not extend to estimation of population-specific phenotypic and ecological traits depending on reporting group structure and trait distribution. Researchers generally assume the trait of interest varies independently from assignment probability, minimizing any bias in trait estimation, even in the presence of mixture composition bias. The current study explores potential violations of that independence when using different analytical models and under different distributions of the trait value and genetic similarity.

Early applications of genetic markers used allozyme loci, whose low diversity largely precluded accurate IA. For that reason, research focused on the development of models that allowed direct estimation of the parameters of primary interest at the time, which was mixture composition in fisheries that included multiple source populations and regions [2], [14]–[16]. The primary advantages of mixture models over IA are twofold. First, mixture models facilitate direct estimation of stock composition proportions, without the need to first estimate population membership through individual assignment (with an unknown degree of estimation error) and then condition on those estimates as if they were real observations in source populations, made without error. Second, all information contained in a mixture sample is utilized by the mixture model, including the true uncertainty in estimation. The resurgent interest in IA was largely fueled by increased power of highly polymorphic microsatellite loci and more recently single nucleotide polymorphisms (SNPs). As genetic baselines continue to improve with the addition of more potential source populations and implementation of more genetic markers, one can expect differences to diminish between mixture model estimates and estimates that condition on preliminary IA. Even so, mixture modeling will continue to enjoy the strongest theoretical foundation and some degree of superior statistical performance as long as uncertainty exists in IA [3], [17].

In this study, we seek the same statistical robustness for ecological and phenotypic trait inference that has been demonstrated for mixture modeling as compared to IA. We extend the Bayesian genetic mixture model to incorporate parameters associated with phenotypic traits. The conditional maximum likelihood (CML) model of Bromaghin et al. [13] is incorporated into a Bayesian framework. Again, the conceptual advantage of this approach is the utilization of all available information in a mixture sample, along with its uncertainty, and minimizing potential biases stemming from conditioning on prior estimates of population membership. The performance of the Bayesian mixture model (BMM) is assessed through comparison with that of IA using the maximum a posteriori (MAP) rule [17]. Under the MAP rule, we assign the individual to the group of source populations for which the posterior source probability is maximal, with application of various minimum probability thresholds.

To achieve the overarching goals above, we consider two diverse example applications, reanalyzing recently published data in light of simulation results. Finally, we make recommendations for specific methods based on their performance under various distributions of the ecological/phenotypic trait and genetic distance among populations. Although we draw heavily on Pacific salmon research, we emphasize the general applicability of our analyses and especially the diversity of genetic and non-genetic traits that can be examined. Our analysis is relevant to all marker classes (allozymes, microsatellite, SNPs, etc.). Applications are limited only by available baseline data for known-origin individuals. We expect increasing interest in our methods as those data become available for more taxa.

## Materials and Methods

We explored two methods of using genetic data to estimate descriptive parameters of phenotypic traits among populations inferred from a genetic mixture. One method, IA, assigned individuals to putative population of origin, using a MAP rule with various probability threshold values to exclude individuals whose origins were less certain. Parameters of interest were then estimated by conditioning on the individual assignments and treating each partitioned subset as an independent sample from the contributing population. The second method utilized a Bayesian mixture model (BMM) to estimate directly the distribution of a phenotypic trait, irrespective of IA. Our study had two elements, first was a test of concept, an analysis of simulated data to inform and direct the re-analysis of two published data sets in the second element of our study. The reanalyzed data sets included fecundity in Yukon River adult Chinook salmon (*Oncorhynchus tshawytscha*) [13], and bacterial kidney disease (BKD) infection prevalence in Puget Sound juvenile Chinook salmon [9]. Originally, a third method was used but was abandoned due to poor performance. We used the posterior source probabilities from the computer package BAYES [2] to weight the trait values observed in individuals (data not shown). We also computed mean trait values of assigned individuals using the MCMC realizations of individual assignment to effectively do the same thing. Either way, we found performance to be inferior to the IA and BMM methods that are the focus of the current study. This was especially true in the case of negative correlation between trait value and genetic similarity (see below).

### Creation of simulated negative and positive correlations between phenotypic trait value and genetic similarity of source populations

To evaluate the performance of the trait inference methods under known experimental conditions, we first applied both methods to simulated data where we could manipulate genetic similarity and trait distribution. To parameterize our model, we used the BKD data set [9] and a portion of the GAPS-Chinook microsatellite baseline [4], but we controlled the level of BKD prevalence among reporting groups. We also manipulated the reporting groups slightly to present a more demanding analytical problem relative to [9]. To do this, we split the Hood Canal and South Sound fall-run reporting group of [9] into two genetically similar reporting groups. The other four reporting groups remained as described in [9], resulting in six reporting groups for our simulations: 1) Fraser and Thompson, 2) Nooksack, 3) Whidbey Basin, 4) South Puget Sound Fall Run, 5) Hood Canal, and 6) South Puget Sound Spring Run (Table 1). The two new reporting groups (4 and 5) are genetically similar and were expected to have a higher degree of misclassification between them, allowing more rigorous evaluation of the IA and BMM methods. We constructed two sets of known BKD infections rates among the six reporting groups, one in which the infection rates were positively correlated with genetic similarity among reporting groups, and a second set which was negatively correlated (i.e., genetically similar populations had dissimilar infection rates). In the positive case, we hypothesized that both methods would perform equally well, as any errors in assignment would be between reporting groups with similar infection rates. In the negative case, however, assignment errors would most likely occur between reporting groups with dissimilar infection rates, which would bias their estimation.

**Table 1:** Populations and reporting groups used as baseline for Puget Sound Chinook salmon individual assignment and mixture modeling for inferring group-specific BKD infection rates.

No.	Population	Reporting group	Group No.
1	Birkenhead	Fraser and Thompson	1
2	Maria Slough	Fraser and Thompson	1
3	Chilliwack	Fraser and Thompson	1
4	Nicola	Fraser and Thompson	1
5	Spius	Fraser and Thompson	1
6	Chilko	Fraser and Thompson	1
7	Nechako	Fraser and Thompson	1
8	Quesnel	Fraser and Thompson	1
9	Stuart	Fraser and Thompson	1
10	Chilcotin	Fraser and Thompson	1
11	Clearwater	Fraser and Thompson	1
12	Deadman	Fraser and Thompson	1
13	Louis	Fraser and Thompson	1
14	Raft	Fraser and Thompson	1
15	Lower Adams	Fraser and Thompson	1
16	Lower Thompson	Fraser and Thompson	1
17	Middle Shuswap	Fraser and Thompson	1
18	Morkill	Fraser and Thompson	1
19	Salmon Fraser	Fraser and Thompson	1
20	Swift	Fraser and Thompson	1
21	Torpy	Fraser and Thompson	1
22	Nooksack	Nooksack	2
23	Lower Sauk	Whidbey Basin	3
24	Marblemount Spring	Whidbey Basin	3
25	Marblemount Summer	Whidbey Basin	3
26	NF Stilliguamish	Whidbey Basin	3
27	Upper Skagit Summer	Whidbey Basin	3
28	Skykomish	Whidbey Basin	3
29	Silliguamish	Whidbey Basin	3
30	Suiattle	Whidbey Basin	3
31	Cascade	Whidbey Basin	3
32	Upper Sauk	Whidbey Basin	3
33	Skykomish H Summer	Whidbey Basin	3
34	Lower Skagit Fall	Whidbey Basin	3
35	Skykomish R Summer	Whidbey Basin	3
36	Upper Sauk R Spring/Summer	Whidbey Basin	3
37	Samish	South Sound Fall	4
38	Snoqualmie	South Sound Fall	4
39	Clear Cr	South Sound Fall	4
40	S Prairie Cr	South Sound Fall	4
41	Soos Cr	South Sound Fall	4
42	Voights Cr	South Sound Fall	4
43	Bear Cr Summer/Fall	South Sound Fall	4
44	Cedar R Summer/Fall	South Sound Fall	4
45	Grovers Cr H	South Sound Fall	4
46	Issaquah Cr Summer/Fall	South Sound Fall	4
47	Nisqually R Summer/Fall	South Sound Fall	4
48	UW H Summer/Fall	South Sound Fall	4
49	George Adams	Hood Canal	5
50	Hamma Hamma	Hood Canal	5
51	NF Skokomish	Hood Canal	5
52	SF Skokomish	Hood Canal	5
53	Hupp Sp	South Sound Spring	6
54	White R H	South Sound Spring	6

A measure of genetic differentiation was computed for each pair of reporting groups. Genetic differentiation between population pairs, *F*_ST_, was calculated with GENEPOP, version 4.1.0 [18]. The average *F*_ST_ among all unique pairs of populations in each pair of reporting groups was computed as a measure of differentiation (Table 2). For the positive case, the two groups with the greatest genetic differentiation (*F*_ST_  = 0.08 between Group 1 and 6) were assigned dissimilar infection rates (0.05 and 0.95, respectively). The rates for the other four population groups were selected to maximize the correlation between the average *F*_ST_ and the difference between reporting group BKD infection rates (e.g., groups 4 and 5 [Table 2]). The opposite process was used to establish infection rates for the negative case. The two most genetically similar reporting groups (4 and 5) were assigned the most dissimilar infection rates (0.05 and 0.95, respectively), and the infection rates for the other four reporting groups were selected to minimize the correlation between the average *F*_ST_ and the difference between reporting group BKD infection rates (Table 2).

**Table 2:** Average pair-wise *F*_ST_ values and BKD infection rates among reporting groups used in the simulations.

Group number						Infection rate	
	2	3	4	5	6	Positive	Negative
1	0.078	0.058	0.071	0.07	0.08	0.05	0.42
2	-	0.035	0.049	0.052	0.075	0.46	0.42
3	-	-	0.026	0.026	0.04	0.69	0.7
4	-	-	-	0.006	0.028	0.81	0.05
5	-	-	-	-	0.025	0.81	0.95
6	-	-	-	-	-	0.95	0.42

**In the positive case, genetically similar groups have similar infection rates, whereas in the negative case genetically similar groups have dissimilar BKD infection rates.**

BKD infection (presence or absence) for a mixture individual was randomly determined by sampling from a Bernoulli distribution with mean infection rate established under either the positive or negative case. We simulated mixtures of 300 individuals with equal proportions (∼16.7%) from each of the six reporting groups, each population within a group contributing equally. We generated 25 replicate datasets for each of the negative and positive cases and analyzed each dataset with IA [1] and BMM [2]. The only condition that differed between the two cases was the correlation (positive and negative) between the genetic and phenotypic trait information. To simulate genetic data for the mixtures, we created single-locus genotypes for each individual by randomly sampling two alleles from the actual baseline, without replacement. Both alleles were then replaced before drawing alleles for the next individual. The sampling wass repeated independently for the 13 loci in the baseline. Data were simulated from 54 baseline populations (Table 1), a subset of the GAPS-Chinook baseline [4].

### Inference of the phenotypic trait from simulated and empirical data

#### Method 1: Individual assignment (IA) with MAP of 0.80 or greater

Method 1 was implemented by genetic mixture analysis software ONCOR [19], [20], which uses conditional maximum likelihood (CML) to assign mixture individuals to the most likely population of origin, based on their genotypes. Probabilities 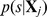 that an individual came from baseline populations are calculated from the relative frequency 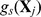
of a fish's genotype 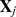
in population *s* and the estimated proportion of population *s* in the mixture sample 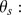


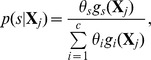
where *c* is the number of populations. ONCOR [19], [20] uses Rannala and Mountain's [1] method to estimate relative genotypic frequencies 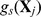 and CML to estimate mixture proportions of contributing populations 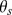. We calculated probabilities of assignment to reporting groups by simply summing individual population probabilities.

The distributions of the phenotypic character among reporting groups were estimated from the mixture individuals assigned to the reporting groups and their measurements of the trait. A mixture individual was assigned to the reporting group for which its probability of assignment was maximal, so long as that probability exceeded a given threshold. Assignments to population of origin were considered uncertain when the probability of assignment fell below the threshold, and those individuals were excluded from further analysis. Origins of assigned fish were assumed without error, and their measurements of the phenotypic character were treated as a representative sample from each reporting group.

To investigate the tradeoff between MAP threshold and potential assignment error, we examined the effect of using values for MAP-rule probability threshold ranging from 0.5 to 0.9. For the analysis of empirical data (see below), we utilized a probability threshold of 0.80. The expectation was that a higher probability threshold would result in more accurate individual assignments but at the cost of reduced sample size because a larger number of fish would have assignment probabilities below the threshold and would be excluded from analysis.

#### Method 2: Bayesian mixture modeling

The BMM was implemented by modifying the BAYES software program [2] to incorporate a novel model for the phenotypic trait in the likelihood of the mixture model [13]. The distributions of the phenotypic character in reporting groups were estimated directly, along with mixture proportions, in a Bayesian framework. As in the likelihood model of Fournier et al [14], we used the phenotypic trait as another character, in addition to the genetic data, to identify the origin of the individual (see below). The BMM analysis involved running multiple independent chains of Markov Chain Monte Carlo (MCMC) samples, simulated draws from the posterior distribution of the unknown parameters, started from diverse parts of the sample space. The first half of each chain was discarded as burn-in, removing dependence on starting values. The Gelman-Rubin shrink factor [21], computed from the second half of each chain, was used to determine convergence of samples to the posterior distribution. A shrink factor near 1.0 and less than 1.2 was taken as evidence of adequate mixing and convergence. Once convergence was achieved, samples from the second half of each chain were pooled and treated as draws from the desired posterior distributions. We used the standard uninformative prior distribution for a normal regression, according to Gelman et al. [21] because we had no prior, independent information regarding the distribution of the unknown regression parameters relating fecundity to fish length. An informative prior could be used if prior, independent information regarding the distribution of the unknowns were available.

### Reanalysis of empirical data – Yukon River adult Chinook salmon fecundity

Bromaghin et al. [13] obtained matched fecundity, length, and microsatellite data (13 GAPS loci) from 403 adult female Chinook salmon collected near the mouth of the Yukon River in Alaska. The study investigated whether Chinook salmon fecundity is in real decline or simply exhibiting temporal variability. Bromaghin et al. [13] extended the CML mixture model by incorporating fecundity parameters for specific population groups. The joint estimation of fecundity and population composition (the population composition was not of interest, per se) avoided potential biases caused by IA errors. Length-adjusted fecundity parameters were estimated for three reporting groups – the Lower, Middle, and Upper Yukon River – composed of 34 individual populations. Their study did not show evidence of a decline in fecundity but rather high interannual variability. Further, length-adjusted fecundity was shown to decrease with increased migration distance to the spawning grounds.

Bromaghin et al. [13] modeled mean fecundity as a normal variate, linearly related to length, and they jointly estimated fecundity parameters and population composition of the mixture. Populations were grouped according to similar fecundity traits rather than genetic similarity. Here we adapted the same model to a Bayesian framework and analyzed the same data for comparison with the CML model of [13]. We replaced the likelihood function for the mixture sample (Equation 6 of [2]) with Equation 5 of [13] so that the probabilities of a mixture individual coming from populations include the probability of fecundity. The BAYES program [2] was modified to accommodate the extended likelihood model, including draws of the unknown regression parameters from the posterior distribution.

Following [13], fecundity of fish *m* (*F_m_*) with mid-eye to tail fork length (*L_m_*) from population group *j* was modeled as a normal variate with mean equal to 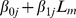 and standard deviation 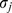. The density was defined as
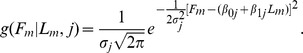



The likelihood model, with notation slightly modified from [13], was:
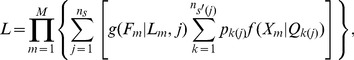
(1)where

*M*  =  number of mixture individuals,


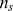
 =  number of reporting groups,


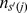
 =  number of populations in reporting group *j*,


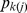
 =  population proportion *k* of reporting group *j*,


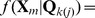
 relative frequency of genotype in population *k* of reporting group *j* given baseline allele frequencies 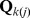, and


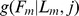
 is defined above.

In addition to the Dirichlet priors for ***p*** and ***Q*** in [2], we used the standard uninformative prior distribution for a normal regression [21; Section 14.2]: uniform on 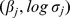 or equivalently, 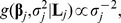 where 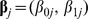 and 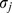 are the regression parameters, and ***L*** *_j_* are the lengths for population group *j*. Following [2], ***p*** and ***Q*** were drawn from Dirichlet posterior distributions. Specifying the uniform prior distribution on 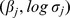 provides that the conditional posterior distribution of 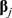 given 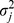
is a normal distribution and that the marginal posterior distribution of 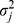
has the form of a scaled inverse- 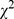 distribution [21; Section 14.2].

### Reanalysis of empirical data – Puget Sound juvenile Chinook salmon BKD

Rhodes et al. [9] examined BKD in juvenile Chinook salmon collected at multiple times and locations in Puget Sound, in the Pacific Northwest of North America. Their study investigated several potential ecological and environmental links with prevalence of this very serious disease. One such link examined was fish origin, determined by coded-wire tags and genetics (13 GAPS microsatellite loci). The authors found that capture location was a better predictor of infection than population origin. Here we examined just the genetic and BKD data from 393 individuals and jointly modeled fish origin and BKD prevalence among populations. Specifically, we estimated infection prevalence rates among the five Puget Sound reporting groups in [9]. The baseline included 54 populations representing the following reporting groups: 1) Fraser and Thompson, 2) Nooksack, 3) Whidbey Basin, 4) South Sound Fall and Hood Canal, and 5) South Sound Spring (Table 1). Note that these are the five originally published reporting groups that differed slightly from the six reporting groups we used in our simulations.

The BKD infection of fish *m* was modeled as a Bernoulli random variable (*d_m_*) with probability 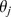 of infection for population group *j*. The density is defined as
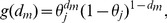
(2)where *d_m_* = 1 if a fish was infected and 0 otherwise. We replaced the normal model of the fecundity-length relationship in Equation 5, with the Bernoulli model above (Equation 6). We used uniform (0,1) prior distributions for the infection rates, resulting in Beta posterior densities for 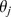 [21]; Section 2.1. The BAYES program [2] was again modified to accommodate the binomial modeling of BKD infection rates.

## Results

### Simulation results: trait inference from IA and BMM

**Figure 1:**
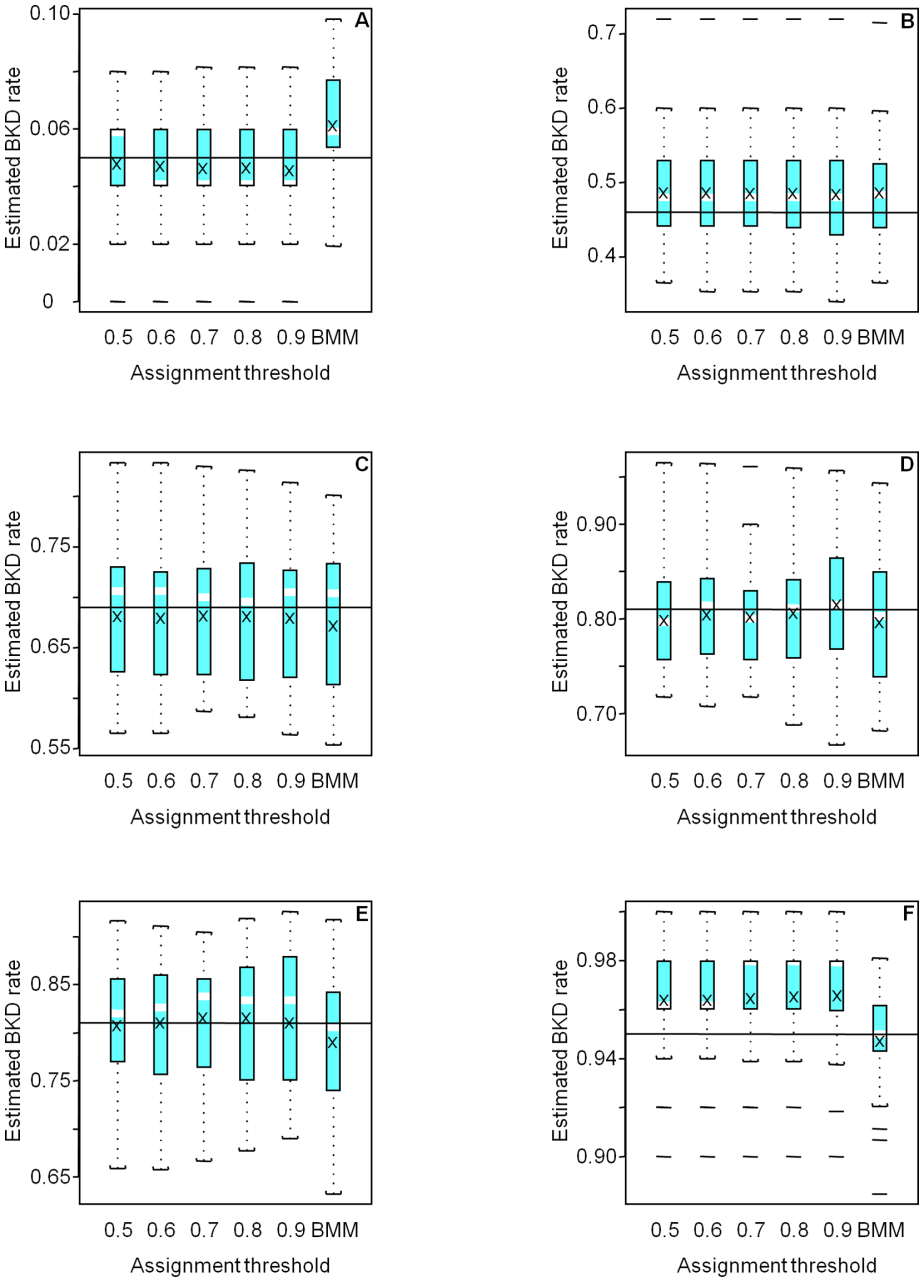
Boxplots of the estimated BKD prevalence rates for the 25 simulations of the positive case in Groups (A) 1, (B) 2, (C) 3, (D) 4, (E) 5, and (F) 6, using the individual assignment method and minimum assignment probability thresholds from 0.5 to 0.9. BMM represents the Bayesian mixture model, X marks the mean estimate. The solid line indicates the true BKD prevalence rate. Note the scale of the y axes vary among panels.

In the positive case, where genetically different Groups 1 and 6 had different BKD prevalence, and genetically similar Groups 4 and 5 had similar BKD rates, the BMM performed similarly to IA. IA estimates for Group 1, with a low BKD value, showed a slight negative bias at all probability thresholds, whereas the BMM slightly over estimated BKD prevalence (Fig. 1A). The opposite was true for Group 6, with a large BKD value. In general, variance was smaller for BMM estimates, but not dramatically so.

**Figure 2:**
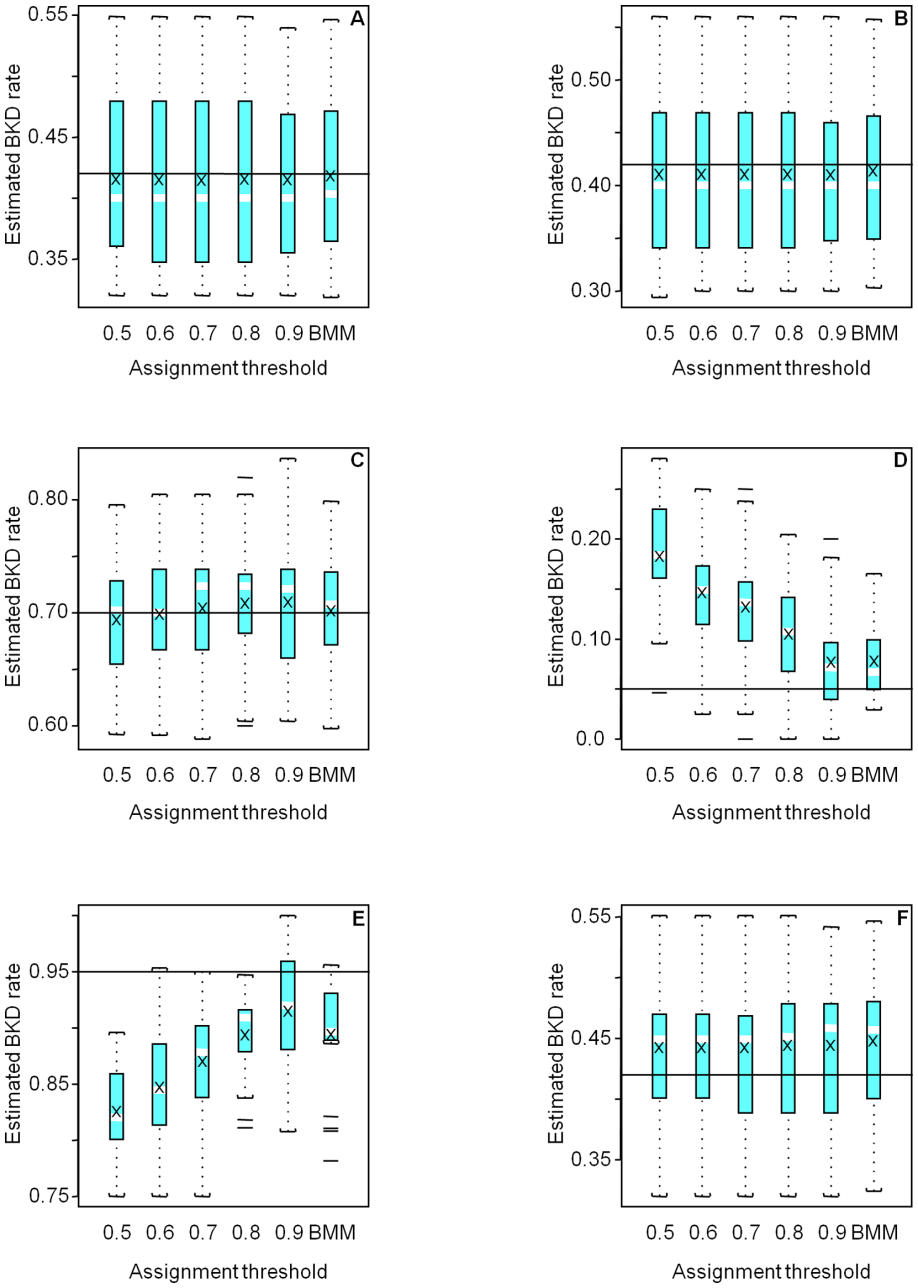
Boxplots of the estimated BKD prevalence rates for the 25 simulations of the negative case in Groups (A) 1, (B) 2, (C) 3, (D) 4, (E) 5, and (F) 6, using the individual assignment method and minimum assignment probability thresholds from 0.5 to 0.9. BMM represents the Bayesian mixture model, and the X marks the mean estimate. The solid line indicates the true BKD prevalence rate. Note the scales of the y axes vary.

**Figure 3:**
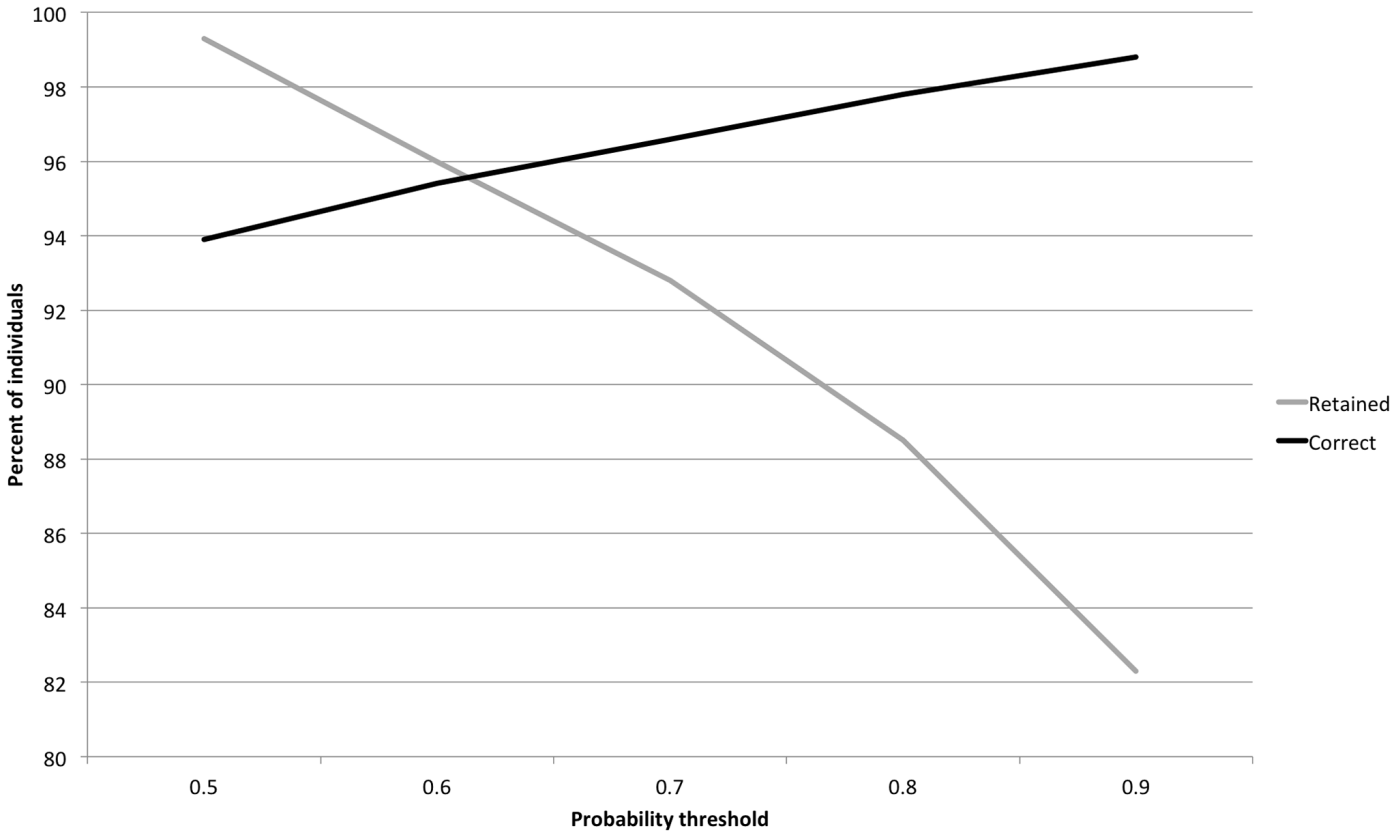
At higher levels of confidence in individual assignment (high posterior probability of group membership), more simulated individuals were correctly classified, but fewer of them met the threshold for inclusion in the analysis, and sample size for trait estimation declined.

Differences were greatest between methods for the negative case, where genetically similar groups 4 and 5 had quite different BKD infection prevalence (0.05 and 0.95, respectively). Both methods over estimated the low BKD prevalence in Group 4 and under estimated the high BKD prevalence in Group 5 (Figs. 2D and 2E). Bias was greatest for IA at MAP probability thresholds below 0.6. Bias improved at higher thresholds, but variance increased in most cases, as more individuals were excluded, and sample size decreased. In general, the BMM gave a favorable balance of accuracy and precision. IA with a high probability threshold (0.9) sometimes gave better accuracy than the BMM but at the cost of decreased sample size (Table 3, Fig. 3) and increased variance.

**Table 3:** The average percentage of individuals in the simulations that were above a minimum assignment probability threshold (and were therefore retained for analysis) and also the average percentage of those individuals that were correctly assigned to each of the groups and to all groups.

	Probability threshold for MAP-rule assignment									
	0.5		0.6		0.7		0.8		0.9	
Group	Retained	Correct	Retained	Correct	Retained	Correct	Retained	Correct	Retained	Correct
1	99.9	99.8	99.8	99.8	99.7	99.8	99.4	99.9	99.2	99.9
2	99.9	99.7	99.6	99.8	99.4	99.9	99.1	99.9	98.5	100.0
3	98.9	95.2	96.6	96.3	94.4	97.5	92.0	98.2	88.2	99.0
4	98.5	83.2	90.0	86.2	81.3	89.0	70.2	92.2	55.1	94.8
5	98.8	85.7	90.1	88.8	82.5	90.9	71.4	93.8	55.0	96.3
6	99.8	99.7	99.7	99.7	99.3	99.8	98.9	99.9	98.0	100.0
All	99.3	93.9	96.0	95.4	92.8	96.6	88.5	97.8	82.3	98.8

### Reanalysis of empirical data – Yukon River adult Chinook salmon fecundity

**Figure 4:**
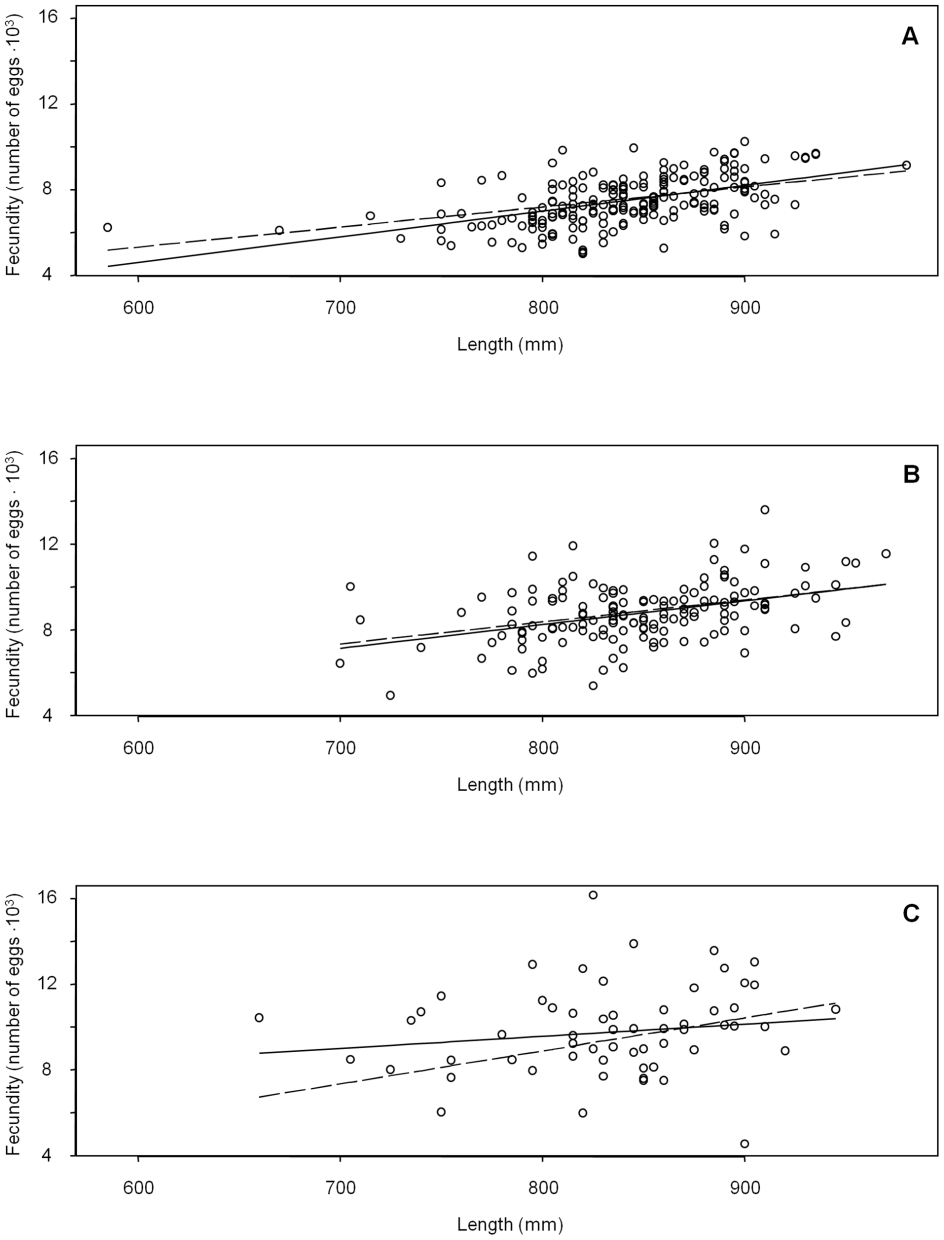
Estimated linear regression models of mean Yukon River Chinook salmon fecundity versus fish length for three reporting groups, (A) Upper, (B) Middle, and (C) Lower, based on the Bayesian mixture model (solid line) and individual assignments (dashed line).

Of 403 mixture individuals, 355 were assigned to one of three reporting groups (Lower, Middle, and Upper Yukon) using a MAP probability threshold of 0.80 or greater, whereas 48 individuals (12%) fell below that probability threshold and were excluded from further analysis. Fecundity estimates were based on the remaining 41, 125, and 189 fish assigned to the Lower, Middle, and Upper Yukon River reporting groups, respectively. For reporting, we fit linear regression models [22] separately for the Lower, Middle, and Upper reporting groups, regressing fecundity on length and assuming normal errors. Estimated slopes, intercepts, standard errors, and 95% confidence intervals are reported in Table 4, and the fitted linear models are plotted in Figure 4.

**Table 4:** Estimated fecundity parameters of intercept (*β_0_*), slope (*β_1_*), and standard deviation (*σ*) for three reporting groups of Yukon River Chinook salmon obtained using individual assignment (IA) and Bayesian mixture modeling (BMM), with standard errors (SE) and either 95% confidence or probability intervals (CI or PI).

Reporting group parameter	IA			BMM		
	Estimate	SE	95% CI	Estimate	SE	95% PI
**Lower**						
*β_0_*	5529	1223	(3055, 8004)	8335	1354	(5614, 10978)
*β_1_*	15.42	4.65	(6.02, 24.82)	5.70	5.32	(−4.71, 16.44)
*σ*	1481			2047	214	(1676, 2518)
**Middle**						
*β_0_*	6125	701	(4739, 7512)	5836	663	(4567, 7149)
*β_1_*	10.28	2.58	(5.18, 15.39)	11.14	2.45	(6.23, 15.83)
*σ*	1443			1319	99	(1146, 1528)
**Upper**						
*β_0_*	5156	404	(4359, 5954)	4413	436	(3546, 5277)
*β_1_*	9.33	1.55	(6.27, 12.39)	11.99	1.65	(8.71, 15.25)
*σ*	1110			994	56	(892, 1111)

Eight chains of 20,000 MCMC samples thinned by 10 were generated using the modified BAYES program. Convergence of the second halves of chains to the posterior distribution was confirmed, and simulated draws from the posterior distributions of the fecundity regression parameters were summarized with a mean and standard deviation for each reporting group (Table 4). Note that Bromaghin et al. [13] imposed additional constraints on the parameters that we did not replicate exactly.

### Reanalysis of empirical data – Puget Sound juvenile Chinook salmon BKD

**Figure 5:**
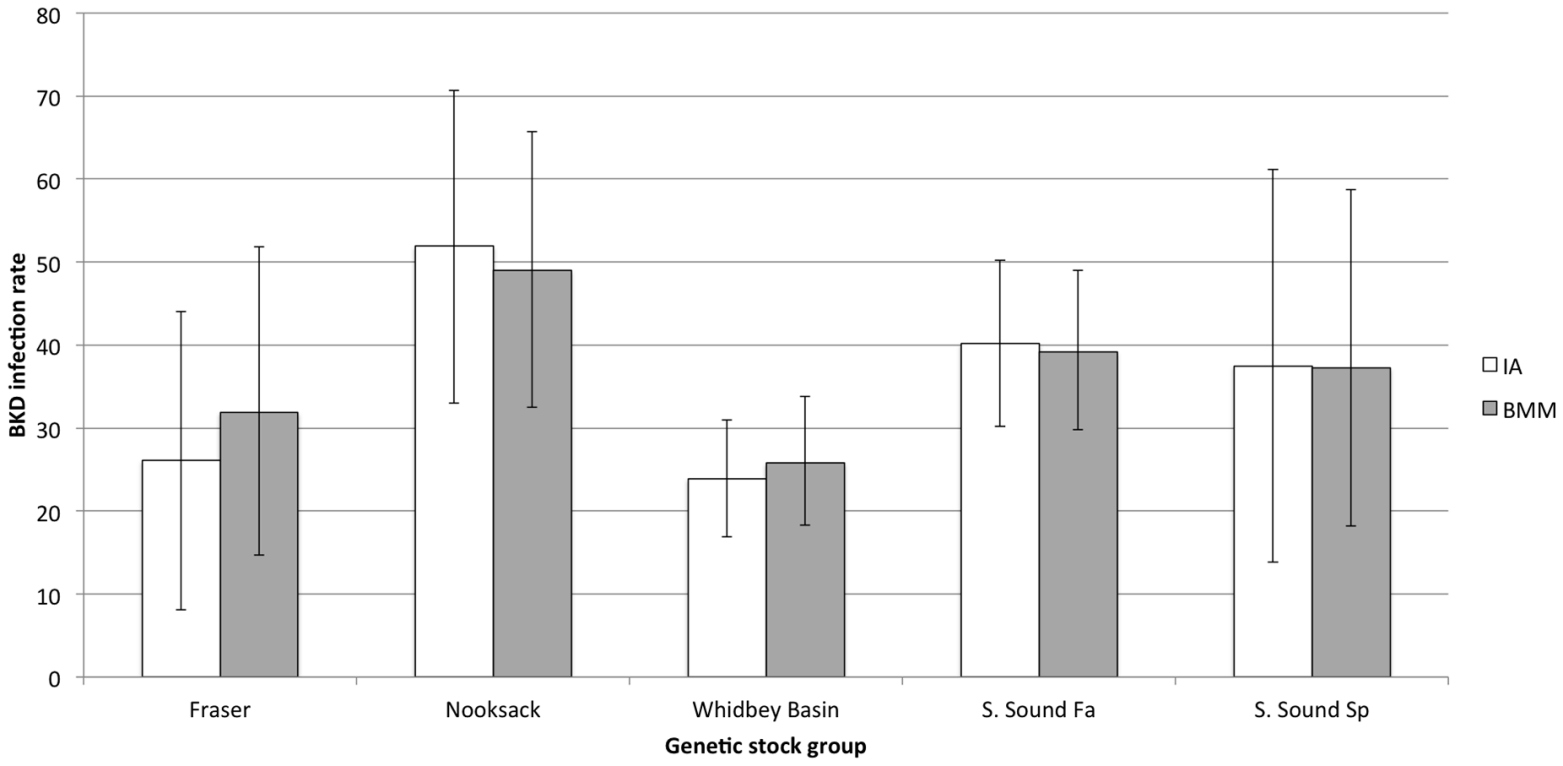
Similar stock-specific estimates of BKD prevalence were obtained using individual assignment (0.8 probability threshold) and Bayesian mixture modeling for reanalysis of Puget Sound Chinook salmon juvenile mixtures. Brackets are shown for confidence and probability intervals (95%).

Of the 373 mixture individuals with BKD data, 73 (20%) did not meet the 0.8 MAP probability threshold and were removed from further analysis. The BKD infection prevalence estimates were based on the remaining 23 fish assigned to the Fraser and Thompson group, 27 to the Nooksack, 142 to Whidbey Basin, 92 to the South Puget Sound fall-run and Hood Canal group, and 16 to South Puget Sound Spring. Infection prevalence rates were calculated as the fraction of fish with BKD assigned to each group (Table 5, Fig. 5). Variances in the rate estimates were calculated from the binomial distribution. Five chains of 20,000 MCMC samples were generated by the modified BAYES program. Convergence of the chains to the posterior distribution was confirmed, and simulated draws from the posterior distributions of infection rates for population reporting groups were summarized with means and standard deviations.

**Table 5:** Estimates of stock-specific BKD prevalence in Puget Sound Chinook salmon population groups from two different methods, with standard errors (SE), and either 95% confidence or probability intervals (CI or PI).

Reporting group	IA^1^			BMM		
	Estimate (%)	SE (%)	95% CI	Estimate (%)	SE (%)	95% PI
Fraser and Thompson	26.1	9.2	(8.1, 44.0)	31.9	9.6	(14.7, 51.8)
Nooksack	51.9	9.6	(33.0, 70.7)	49.0	8.5	(32.5, 65.7)
Whidbey Basin	23.9	3.6	(16.9, 31.0)	25.8	4.0	(18.3, 33.8)
South Sound Fall and Hood Canal	40.2	5.1	(30.2, 50.2)	39.2	4.9	(29.8, 49.0)
South Sound Spring	37.5	12.1	(13.8, 61.2)	37.2	10.4	(18.2, 58.7)

1 300 of 373 individuals satisfied the 0.8 MAP threshold and were included in the trait inference analysis (20% discarded).

## Discussion

In both example applications that re-analyzed published Chinook salmon data, we obtained similar estimates for the group-specific phenotypic traits from both IA and BMM. Our estimates were also comparable to those previously reported [9], [13]. The greatest difference between the two sets of estimates in both examples involved the fecundity parameters for the Lower Yukon River reporting group (Table 4). Although the IA and BMM confidence/probability intervals for the three Lower reporting group parameters also overlap, the magnitude of the differences between the point estimates appears biologically meaningful. Bromaghin et al. [13] hypothesized that the similar differences they observed were caused by assignment error between genetically similar populations having dissimilar fecundity traits, which is analogous to the conditions we established in the negative-case simulations where BMM outperformed IA. The similarity between the IA and BMM estimates in the BKD example is likely attributable to the power of the GAPS-Chinook baseline to assign individuals to source populations with high accuracy, especially when a MAP probability threshold is employed (Table 3).

Our analysis of simulated data suggested that for simple mixture problems, such as a large mixture sample including only a few genetically distinct source populations, both IA and BMM perform similarly. This was especially true if the trait was positively correlated with genetic similarity among reporting groups. BMM provided slightly better accuracy and precision when the trait was negatively correlated with genetic similarity. In general, however, IA with a 0.8 MAP threshold performed better than expected. We suspect the favorable performance of IA observed here is attributable to a combination of the 0.8 MAP threshold maintaining adequate sample sizes and accuracy of IA based on the GAPS-Chinook microsatellite baseline.

IA for trait inference does not necessarily suffer the same bias as IA for population mixture composition estimates [17]. In estimating mixture compositions where a MAP rule is employed with a threshold for IA, individuals from reporting groups that are not genetically distinct will more likely be removed from the analysis because they fractionate their posterior probability among multiple potential source populations, and their maximum assignment probability is more likely to fall below the threshold. Thus, the contributions of reporting groups that are less distinct can be under estimated in the mixture. Whether this bias affects estimates of population-specific traits depends on whether the trait is negatively or positively correlated with genetic similarity among reporting groups. In the positive case, trait estimates from IA are expected to be unbiased. Even though we might underestimate the true number of fish from genetically similar reporting groups, we are unlikely to end up with a biased set of individuals relative to the traits they express. Precision might suffer from diminished sample size, but trait estimates should not be biased in the same way that population mixture composition can be [17].

Although bias was not necessarily expected in comparing IA and BMM (at least not where the trait is positively correlated with genetics), our results serve to underscore a classical problem in biological sampling – the need to balance accuracy of IA with sample size and therefore statistical power. On one hand, IA with a high MAP-rule threshold will better assure that individuals included in the analysis are accurately classified. On the other hand, if mixture samples are small to begin with, as they often are in ecological studies, then the number of individuals with assignment probabilities above the MAP threshold can be quite small. If one lowers the threshold to include more individuals, assignment error and therefore trait estimation bias can increase. This is not such a problem if the trait distribution is correlated with the genetic structure of reporting groups (genetically similar groups have similar mean trait values), but if genetically similar reporting groups have very different trait values, then clearly misassignments will bias estimates of population-specific traits. We saw signs of such bias in our simulation studies where we created trait distributions having both positive and negative correlation with genetic distance. The genetic data we used were based on a regional subset of the GAPS-Chinook microsatellite baseline. The 13 GAPS loci are highly variable, with nearly 500 alleles coast wide and highly significant allele frequency differences among populations and among regions [4]. This diversity provided significant power to accurately assign individual Chinook salmon to their putative population of origin. The difference in performance between IA and BMM would be greater in applications with less powerful baselines, which could be caused by fewer, less variable loci, low differentiation among populations, or because mixture samples are degraded and provide quality genotypes for only a subset of loci. In many ecological genetic studies of the type we consider here, power is often limited by small mixture samples. Genetic reporting groups that have small contributions to a mixture may provide very few individuals from which to estimate trait parameters. In our simulations, a MAP threshold of 0.8 seemed a good compromise between assignment accuracy and sample size, which contribute to precision and accuracy in trait estimation (see also [10]).

Potential applications of our method are extremely broad, as has been noted previously [13]. Almost any population-specific phenotypic or ecological trait can be inferred using the methods we describe. Many ecological, management, and conservation problems involve consideration of individual populations, geographic regions or groups of populations. Habitat restoration efforts often have monitoring and evaluation elements that seek to document the use of restored habitats by populations targeted for recovery [23]. Ecotoxicology studies need to determine if different populations with different migratory routes have different body burdens of toxic compounds [6]. Our results show that the relatively simple IA approach with a MAP probability threshold can often provide adequate estimates for many ecological applications. In more demanding trait inference problems, use of a BMM would be prudent, where, for example, mixtures are small or reporting groups are genetically similar. BMM should always work as well or better than IA on average and does not require an arbitrary decision to discard some portion of the data (MAP probability threshold). We showed that BMM is expected to be most useful when the composition of reporting groups and the distribution of the trait do not closely align with the genetic structure among populations.

In practical application of mixture analysis, decisions must be made regarding the structure of the reporting groups. Mixture allocations are made to individual populations and those allocations (or individual assignments) with their associated trait values are combined to estimate the trait values for the reporting groups. Two considerations are important to this application, 1) different levels of genetic similarity among populations within reporting groups, and 2) potential variation in the trait of interest among populations within reporting groups. These considerations reflect a fundamental challenge of natural resource management and especially of fisheries. Rarely do management units fully align with population genetic structure. Genetically similar units sometimes have different trait values and must be managed separately. BMM tends to be more robust to these complications than IA [13], [17].

## Conclusions

We compare the performance of two available methods, individual assignment and Bayesian mixture models, for investigating the distribution of phenotypic traits among populations by using a single mixture sample and population-specific genetic baseline data. The most attractive feature of IA is its ease of implementation using available software (e.g., ONCOR, GeneClass, etc.). Moreover, despite theoretical shortcomings, it performs well under a range of conditions in which the number of individuals assigned to each group is adequate and assignment errors are relatively rare between reporting groups. As a precaution, we recommend careful assessment of the power of a baseline to accurately assign individuals to reporting groups that are of research or management interest. While the performance of BMM equals or exceeds that of IA, the former is more difficult to implement, and software is not available that is capable of handling the diverse array of traits that may be of interest. However, if a preliminary IA analysis indicates that the trait of interest appears negatively correlated with genetic similarity among populations, then the BMM approach is probably essential to provide minimally biased group-specific trait inference. If these situations are common, then additional effort may be warranted to develop custom software solutions.
